# Interventions to decrease the risk of adverse cardiac events for post-surgery or chemotherapy patients taking serotonin (5-HT3) receptor antagonists: protocol for a systematic review and network meta-analysis

**DOI:** 10.1186/2046-4053-2-45

**Published:** 2013-06-28

**Authors:** Andrea C Tricco, Charlene Soobiah, Jesmin Antony, Brenda Hemmelgarn, David Moher, Brian Hutton, Sharon E Straus

**Affiliations:** 1 Li Ka Shing Knowledge Institute, St. Michael’s Hospital, 209 Victoria Street, East Building, Toronto, ON M5B 1 T8, Canada; 2 Departments of Medicine and Community Health Sciences, University of Calgary, 2500 University Dr NW, Calgary, AB T2N 4Z6, Canada; 3 Clinical Epidemiology Program, Centre for Practice-Changing Research, Ottawa Hospital Research Institute, 725 Parkdale Ave., Ottawa, ON K1Y 4E9, Canada; 4 Department of Geriatric Medicine, University of Toronto, 27 Kings College Circle, Toronto, ON M5S 1A1, Canada

## Abstract

**Background:**

Patients undergoing surgery or chemotherapy often experience nausea and vomiting. To increase their quality of life and treatment satisfaction, antiemetic medication, such as serotonin receptor antagonists, is often prescribed for patients experiencing these symptoms. However, early warning signs suggest that serotonin receptor antagonists can cause harm, including arrhythmia. Our objective is to identify the most effective interventions that mitigate the risk of adverse cardiac events associated with serotonin receptor antagonists in patients undergoing surgery and chemotherapy through a systematic review and network meta-analysis.

**Methods/design:**

We will search electronic databases (for example, MEDLINE, Embase) from inception onwards, as well as dissertations and governmental reports, to identify interventions (for example, telemetry, electrocardiography, electrolyte monitoring) that decrease the cardiac risk associated with serotonin receptor antagonists among surgery and chemotherapy patients. Eligible comparators include placebo or supportive care; eligible study designs are experimental studies (randomized controlled trials (RCTs), quasi-RCTs, non-RCTs), non-experimental studies (interrupted time series, controlled before-and-after studies), and cohort studies. Outcomes of interest include arrhythmia, sudden cardiac death, QT prolongation, PR prolongation, and all-cause mortality. We will include unpublished studies and studies published in languages other than English.

Draft inclusion and exclusion criteria will be established and pilot tested amongst the team. Subsequently, two team members will screen the results in duplicate and resolve conflicts through discussion. The same process will be followed to screen full-text articles, data abstraction, and appraise quality or risk of bias. To determine validity of results, experimental and quasi-experimental studies will be assessed using the Cochrane Effective Practice and Organisation of Care (EPOC) Risk of Bias tool, while cohort studies will be appraised using the Newcastle-Ottawa Scale. We anticipate sufficient data and homogeneity to conduct random effects meta-analysis and network or mixed treatment comparisons meta-analysis, if appropriate.

**Discussion:**

Our results will provide information regarding the utility of different strategies that can be used to mitigate cardiac risk amongst patients taking serotonin antagonist receptors. Such results are likely to be of use to clinicians prescribing these agents, as well as policy makers responsible for making decisions about antiemetic medications.

**Trial registration:**

PROSPERO registry number: CRD42013003565

## Background

Many patients undergoing chemotherapy or surgery experience nausea and vomiting [1,2]. These symptoms are not only distressing to patients, but may cause adverse events, such as an increased length of hospitalization [3], pulmonary complications, and wound dehiscence [4]. To prevent these symptoms, many patients are prescribed serotonin (5-HT3) receptor antagonists.

Serotonin receptor antagonists are powerful antiemetic medications that inhibit nerves in the gastrointestinal tract, blocking the emetic reflex [5]. Ondansetron (brand name Zofran), dolasetron (brand names Anzemet, Anemet), and granisetron (brand names Sancuso, Kytril, Kevatril) are first-generation 5-HT3 receptor antagonists, while palonosetron (brand names Aloxi, Alexi) is a second-generation receptor antagonist [6].

Although 5-HT3 receptor antagonists are effective for preventing nausea and vomiting among patients undergoing chemotherapy or surgery [1,7-9], early warning signs suggest that these agents might cause cardiac harm. For example, two studies examining chemotherapy among children found that 5-HT3 induced prolongation of the QT interval [10,11]. In the first study, the QT interval was increased up to 24 hours after the antiemetic was given, but this was asymptomatic and serious arrhythmias were not noted [10]. In the second study, a similar transient increase in the QT interval was observed, but was not found to be clinically significant [11]. In this study, the prolonged QT interval was associated with the 5-HT3 receptor antagonist granisetron but not with ondansetron [11]. The relationship between 5-HT3 receptor antagonists and cardiac risk has not been confirmed by systematic review.

As a result of these concerns, regulatory actions have been taken against these agents in some countries. For example, dolasetron is contraindicated for any use in children and for postoperative nausea and vomiting in adults in Canada [12]. If this association is found to be valid, it might be important to consider interventions that might mitigate this risk.

Several diagnostic tests exist for monitoring or mitigating cardiac risk. These include electrocardiography, electrolyte monitoring and replacement, and adjustment of concomitant antiarrhythmics. Electrocardiography can be employed after surgery or post-chemotherapy and provides information on PR prolongation, which might be indicative of arrhythmic events and all-cause mortality [13]. Electrocardiography can also detect QT prolongation, which has been associated with arrhythmic events, including torsades de pointes tachycardia [14], and sudden death [15]. Continuous electrocardiography (>24 hours) can be achieved using a cardiac telemetry monitor [16]. Imbalance in electrolytes, including hypokalemia, hypomagnesemia, and hypocalcemia, might lead to QT interval prolongation, suggesting the implementation of electrocardiography [17]. Finally, the use of antiarrhythmics among patients with cardiac abnormalities [18] might be a viable option for patients administered 5-HT3 receptor antagonists who experience cardiac harm.

These interventions can be implemented amongst patients administered 5-HT3 receptor antagonists for nausea and vomiting. However, such interventions would inflict cost to the system and burden to patients. Our objective is to determine whether interventions can be implemented to mitigate the risk of adverse cardiac events associated with 5-HT3 receptor antagonists amongst surgery and chemotherapy patients through a systematic review and network meta-analysis. This ‘query’ was posted by policy makers in Canada, and our results will inform their decision making for these agents.

## Methods/design

Our systematic review protocol was compiled, reviewed by the team, and peer reviewed by systematic review methodologists and pharmacoepidemiologists. It was then registered with the PROSPERO database (CRD42013003565). The reporting of our review is based on guidance from the Preferred Reporting Items for Systematic reviews and Meta-analyses Protocols (PRISMA-P) [19]. We have submitted a protocol to a complementary review on this topic to *Systematic Reviews* (Tricco *et al*., personal communication). Therefore, the methods will only be described briefly here.

### Eligibility criteria

Our eligibility criteria will be based on the PICOS criteria (patients, interventions, comparators, outcomes, study designs), outlined in Additional file 1:

•Patients: studies of patients of all ages receiving 5-HT3 antagonist receptors for nausea and vomiting symptoms post-surgery or after chemotherapy will be included. Studies on all forms of chemotherapy will be included, as well as studies on patients who are chemotherapy-naïve or have received chemotherapy previously.

•Interventions: strategies to mitigate cardiac risk amongst these patients, such as electrocardiography, telemetry, adjustment of antiarrhythmics, and electrolyte monitoring and replacement, will be included.

•Comparators: placebo or supportive care will be eligible comparators.

•Outcomes: the primary outcome is arrhythmia and secondary outcomes are sudden death, QT prolongation, PR prolongation, all-cause mortality, nausea, and vomiting.

•Study designs: we will include experimental studies (randomized controlled trials (RCTs), quasi-RCTs, non-RCTs), quasi-experimental studies (interrupted time series, controlled before-and-after studies), and cohort studies.

•Other limitations: study inclusion will not be limited by publication status, language of dissemination, duration of follow-up, or period of study conduct.

### Information sources and literature search

To identify relevant literature, we will search MEDLINE, Embase, and Cochrane Central from inception onwards. The electronic literature search will be supplemented by searching for unpublished and difficult-to-locate material [20], such as public health websites, trial registers, and guideline producer websites. We will also scan the references of included studies, contact 5-HT3 manufacturers, and contact prolific authors in the field.

An experienced librarian will draft the search strategies. This will subsequently be peer reviewed by another expert librarian using the Peer Review of Electronic Search Strategies (PRESS) checklist [21]. The draft literature search for the main search strategy has been presented in a protocol of a complementary review on this topic (Tricco *et al*., personal communication).

### Study selection process

A calibration exercise will be conducted by the team using the draft eligibility criteria on a random sample of 50 titles and abstracts from the literature search. The eligibility criteria will be revised, as necessary. Subsequently, two team members will screen the citations in duplicate. Conflicts will be resolved by team discussion. The same process will be followed for full-text screening.

### Data items and data collection process

Following a similar process to screening the citations, two team members will abstract the following data in duplicate:

1. Study characteristics, such as setting, country where the study was conducted, details on the 5-HT3 medications, comparator used, and type of test conducted to assess cardiac risk.

2. Patient characteristics, such as mean age, percent women, type of surgery, and type of cancer.

3. Outcome results, such as number of patients experiencing arrhythmia, and mean and standard deviation for PR prolongation.

We will ensure that companion reports are sorted and will contact authors for data clarification.

### Methodological quality and risk of bias appraisal

Studies will be assessed using study-design specific tools. Experimental and quasi-experimental studies will be appraised using the Cochrane Effective Practice and Organisation of Care (EPOC) Risk of Bias Tool [22], while cohort studies will be assessed using the Newcastle-Ottawa Scale [23]. Lastly, publication bias will be visually assessed using funnel plots [24].

### Synthesis of included studies

First, we will describe our results from the data obtained through our data abstraction. Second, we will attempt to conduct a meta-analysis using a random effects model [25] in SAS Version 9.2 [25]. Meta-regression analysis will be conducted if the data are homogeneous, as per an *I*^2^ statistic of at least 60% [26]. Studies will be analyzed separately by age group (children versus adults) and patient population (surgery versus chemotherapy). Third, network meta-analysis will be conducted in WinBUGS [27], if feasible. Consistency of results will be conducted by comparing the results of our frequentist meta-analysis with those obtained from the network meta-analysis, using methods described elsewhere [28,29]. Sensitivity analysis will be conducted to explore the effect of risk of bias and quality (for example, low versus high risk of bias), attrition rates (for example, low versus high attrition), 5-HT3 dosage and formulations, inclusion of quasi-experimental and cohort studies, and priors used in the Bayesian meta-analysis [30] on our results.

## Discussion

This is the first systematic review that we are aware of to focus specifically on strategies to mitigate cardiac risk amongst patients undergoing surgery or chemotherapy who are administered 5-HT3 receptor antagonists. If effective interventions exist, patients, clinicians, and policy makers will have to weigh the pros and cons of using 5-HT3 medication. For example, some patients might wish to forgo the use of these antiemetic medications if they are informed that this might lead to ongoing monitoring, or perhaps patients who are already at risk of cardiac harm should not be prescribed 5-HT3 antagonist receptors. However, patients will not be fully informed about this until the utility of these mitigation strategies is determined.

We will use numerous strategies to diffuse our research results. Examples include conference presentations, open access journal publications, user-friendly executive summaries, and dissemination meetings with patients, healthcare providers, and policy makers. We will also consider dissemination through social media tools, such as Twitter and facebook.

## Abbreviations

5-HT3: Serotonin; AHRQ: Agency for Healthcare Research and Quality; EPOC: Evidence Practice and Organisation of Care; PICOS: Patients, interventions, comparators, outcomes, study designs; PRESS: Peer Review of Electronic Search Strategies; PRISMA-P: Preferred Reporting Items for Systematic Reviews and Meta-Analyses Protocol; RCT: Randomized controlled trial.

## Competing interests

The authors report no conflict of interests.

## Authors’ contributions

ACT conceived the study, designed the study, helped obtain funding for the study, and helped write the draft protocol. CS registered the protocol with the PROSPERO database and edited the draft protocol. JA edited the draft protocol. BH, DM, and BH helped conceive the study, and edited the protocol. SES conceived the study, designed the study, obtained the funding, and helped write the draft protocol. All authors read and approved the final protocol.

## Supplementary Material

Additional file 1 Draft Eligibility Criteria. Click here for file
