# Supplementary material for: Interventions to decrease the risk of adverse cardiac events for post-surgery or chemotherapy patients taking serotonin (5-HT3) receptor antagonists: protocol for a systematic review and network meta-analysis
Source: Syst Rev. 2013 Jun 28;2:45. doi: 10.1186/2046-4053-2-45 (PMC3701482; doi:10.1186/2046-4053-2-45)
Supplement: Additional file 1 — Draft Eligibility Criteria. [file 2046-4053-2-45-S1.docx]

**Appendix: Draft Eligibility Criteria**

Level 1 screening

1. Does this study include adult patients (aged ≥ 18 years) or children who are undergoing chemotherapy or surgery?

YES____ NO____ UNCLEAR____

1. Is this an experimental, quasi-experimental or cohort study?

YES____ NO____ UNCLEAR____

1. Are patients treated with the following 5-HT3 receptor inhibitors?

YES____ NO____ UNCLEAR____

| **Generic name** | **Trade name(s)** |
| --- | --- |
| Ondansetron | Zofran |
| Dolasetron | Azemet, Anemet |
| Granisetron | Sancuso, Kytril, Kevatril |
| Palonosetron | Aloxi, Alexi |

1. Does the study compare a 5-HT3 receptor inhibitor with placebo or supportive care?

YES____ NO____ UNCLEAR____

1. Does the study examine the use of interventions to mitigate cardiac risk (e.g., telemetry, ECG monitoring, adjustment of antiarrhythmics, electrolyte monitoring and replacement)?

YES____ NO____ UNCLEAR____

- If you answer NO to any of these questions, the citation/study will be excluded. All othercitations will be included.Level 2 screening

1. Does this study include adult patients (aged ≥ 18 years) or children who are undergoing chemotherapy or surgery?

YES____ NO____ UNCLEAR____

1. Is this an experimental, quasi-experimental or cohort study?

YES____ NO____ UNCLEAR____

1. Are patients treated with the following 5-HT3 receptor inhibitors?

YES____ NO____ UNCLEAR____

| **Generic name** | **Trade name(s)** |
| --- | --- |
| Ondansetron | Zofran |
| Dolasetron | Azemet, Anemet |
| Granisetron | Sancuso, Kytril, Kevatril |
| Palonosetron | Aloxi, Alexi |

1. Does the study compare a 5-HT3 receptor inhibitor with placebo or supportive care?

YES____ NO____ UNCLEAR____

1. Does the study examine the use of interventions to mitigate cardiac risk (e.g., telemetry, ECG monitoring, adjustment of antiarrhythmics, electrolyte monitoring and replacement)?

YES____ NO____ UNCLEAR____

1. Does the study report at least one of the following outcomes?

Arrhythmia, sudden cardiac death, QT prolongation, PR prolongation, all-cause mortality, nausea, or vomiting.

YES____ NO____ UNCLEAR____

- If you answer NO to any of these questions, the citation/study will be excluded. All other full-text articles will be included.
